# Evaluation of the physical, antioxidant, and organoleptic properties of biscuits fortified with edible flower powders

**DOI:** 10.1002/fsn3.3993

**Published:** 2024-02-01

**Authors:** Beatrix Sik, Zsolt Ajtony, Erika Lakatos, Laura Hanna Gál, Rita Székelyhidi

**Affiliations:** ^1^ Department of Food Science Albert Kázmér Faculty of Agricultural and Food Sciences of Széchenyi István University in Mosonmagyaróvár Mosonmagyaróvár Hungary

**Keywords:** antioxidant properties, biscuit fortification, consumer acceptance, edible flowers

## Abstract

Foods enriched with plants have gradually become an area of increasing research interest because plant ingredients may offer several positive effects on human health and the body. The aim of our study was to develop biscuits fortified with three different types of edible flowers (marigold, lavender, and rose) and examine their physical and antioxidant characteristics, as well as consumer acceptability. The antioxidant properties and characterization of biscuits highlighted that edible flowers may cause increased total polyphenol and total flavonoid yields, as well as DPPH radical scavenging activities. Concerning biscuits fortified with rose petals, the total monomer anthocyanin content was also raised. In addition, the results showed that the antioxidant properties of biscuits increased with increasing concentration (from 2.5% to 5.0%) of edible flowers. Despite this, the consumer acceptability results clearly showed that the addition of marigold and lavender at a concentration of 5.0% caused significantly decreased overall acceptance. We found that the fortification step may increase the spread ratio, which is an important quality attribute of biscuits. We found slight variations in the diameter, thickness, and baking loss parameters of fortified biscuits compared to the control. All in all, the best results were obtained when the biscuits were fortified with rose petals at a concentration of 5%.

## Graphical abstract

Nowadays, people pay more attention to health and nutrition. Therefore, it is important to supplement products made only from wheat flour, which is known to be a less good choice compared to whole meal products, with valuable ingredients to make them healthier. According to this, the aim of our study was to assess the effect of replacing a proportion of wheat flour with edible flower powders on the physical and antioxidant properties of biscuits and their acceptability to consumers.

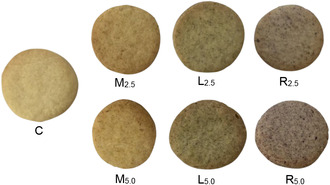

## 1 INTRODUCTION

In recent years, the food industry has paid increasing attention to compounds with antioxidant properties because of their beneficial physiological effect. Phenolic compounds are secondary plant metabolites that can be classified into several subgroups, including phenolic acids, flavonoids, stilbenes, and others. They can improve brain function and blood sugar levels, and protect against cancer and heart disease, among other benefits (Zhang et al., 2022). Plants are also rich in other antioxidant compounds such as vitamins. Edible flower petals are good sources of vitamins A, C, and E. These vitamins are well known for their anti‐inflammatory, immune‐stimulatory, or neuroprotective effects (Skrajda‐Brak et al., 2020).

Edible flowers are often used by chefs around the world as flavoring ingredients in salads, soups, desserts, or drinks for their fresh and exotic aromas. At the same time, the latest research has already confirmed that these plants are rich in several bioactive compounds, like herbs, so they can have positive beneficial effects on the human body and health (Yasar et al., 2022). The most abundant biologically active compounds found in edible flowers are phenolic acids, flavonoids, and anthocyanins. For instance, the main phytochemicals (lutein, flavonoids) of marigold have free radical‐scavenging and cytotoxic effects against various cancerous cells. In addition, scientists have shown that anthocyanin compounds (cyanidin 3,5‐di‐O‐glucoside, pelargonidin 3,5‐di‐O‐glucoside) found in rose petals may play a critical role in the prevention of diabetes (Kumari & Ujala, 2021). For this reason, edible flowers seem to be a promising raw material for the formulation of functional foods (Jadhav et al., 2023). The term functional foods is used to describe healthy foods or food components, which offer benefits beyond adequate nutritional effects and may improve health (Duttaroy, 2019). Currently, a number of studies have been published where these plants were added to various foods, including yogurt (Escher et al., 2019; Pires et al., 2018; Qiu et al., 2021), bread (Podgórska‐Kryszczuk & Pankiewicz, 2023), cookies (Hnin et al., 2020), or chicken meat nuggets (Madane et al., 2019). Recent studies have shown that there is a need to move beyond the more traditional interpretation (e.g., decoration) of the role of flowers in the kitchen. Some artificial food additives are currently the topic of debate because of the serious health risks associated with them. Natural colorants are safe because they have no harmful side effects compared to artificial colorants. According to the color parameter studies of yogurt enriched with different types of flowers, Pires et al. (2018) found that rose extract is the most suitable alternative to E163 (anthocyanin extract) for the coloring of yogurts. However, many flowers cannot be used as coloring agents because of their color, but they have a lot of potential. For instance, Madane et al. (2019) showed that the addition of Moringa flower (white color) to chicken meat nuggets can improve their quality and reduce lipid peroxidation during cooking. Loizzo et al. (2015) investigated the potential antioxidant activities and the hypoglycemic activities of different edible flowers. Their study highlighted that *S. nigra* flower extract exhibits good antioxidant potential, while *M. sylvestris* has a very promising hypoglycemic activity in comparison with the commercial drug acarbose.

Biscuits are popular as a cereal snack food all over the world, particularly among children. These are typically small, sweet, or savory products made from wheat flour. As people become more conscious of health and nutrition, it is important to supplement products made only from wheat flour with valuable ingredients to make them healthier (Yadav et al., 2022). According to this, the aim of our study was to assess the effect of replacing a proportion of wheat flour with edible flower powders on the physical and antioxidant properties of biscuits and their acceptability to consumers.

## 2 MATERIALS AND METHODS

### 2.1 Chemicals and raw materials

The Folin–Ciocalteu reagent, gallic acid, sodium acetate, absolute ethanol, potassium chloride, n‐hexane, and formic acid were bought from Merck. 2,2‐diphenyl‐1‐picrylhydrazyl (DPPH), aluminum chloride, quercetin hydrate, and L‐ascorbic acid were purchased from Sigma‐Aldrich. Anhydrous sodium carbonate was supplied by Riel‐de Haën. Methanol was provided by Reanal, while hydrochloric acid was supplied by Biolab. High‐purity deionized water (18 MΩ cm) was generated by a Zeener Power I (Human Corporation, Korea) system. All the raw materials were food grade. The flour (BL55, Belbake), powdered sugar (Koronás), baking powder (Belbake), margarine (Rama), and milk (Pilos) were purchased from local supermarkets.

### 2.2 Preparation of edible flower petal flour

While the rose petals and lavender flowers were from our own cultivation (Nárai, Vas County, Hungary; coordinates: 47°10′N, 16°45′ E), the marigold petals were purchased from a local shop (Naturland, Herbaház, Hungary). Both rose petals (June) and lavender flowers (July) were harvested in full bloom from 2‐year‐old bushes. The bushes were planted on brown earth soil and no chemical spraying was used during cultivation. Afterward, the samples were air‐dried in a place protected from sunlight for 1 week. During the drying process, the average temperature and relative humidity were 25 ± 3°C and 54 ± 2%, respectively. The dried plants were ground into flour with an electric chopper blender (MQ20WH, Braun, Germany) and immediately used to prepare biscuits. The average moisture content of all dried flower material was around 8.6%.

### 2.3 Biscuit preparation

**FIGURE 1:**
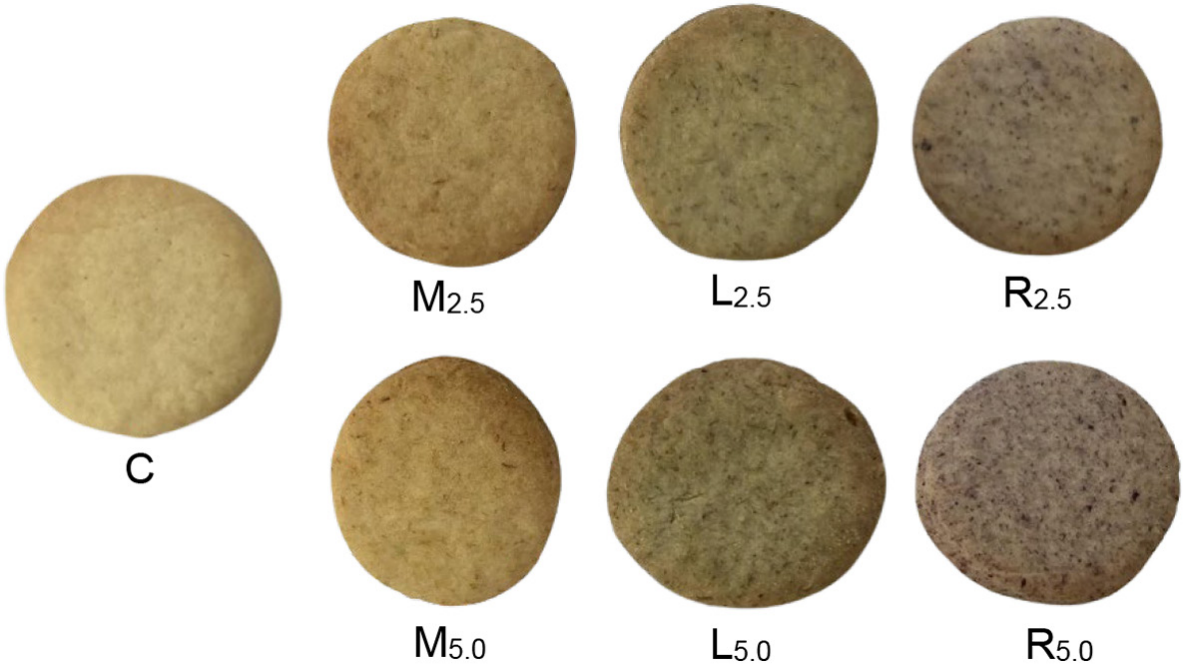
The prepared biscuits after baking.

The biscuit preparation process consisted of two steps. In the first step, 120 g flour, 37 g powdered sugar, 3 g baking powder, and 65 g margarine were combined and mixed. Then, milk (12 g) was added and worked through to form a dough (control: C). In the case of fortified products, grounded edible flower petals (marigold: M; lavender: L., rose: R) were incorporated into biscuits at two levels (2.5% and 5.0%) by replacing an equivalent amount of flour. All prepared dough was rolled out to a thickness of 0.5 cm and cut into rounds. The biscuits were baked (H2561 B, Miele, Germany) for 15 min at 170°C (Figure 1).

### 2.4 Extraction of biscuit samples

For the extraction, the samples were ground into a fine powder using a mortar and pestle. An aliquot (3 g) of the homogenized sample was defatted with 30 mL of n‐hexane by vortexing for 1 min and sonication for 5 min. Afterward, samples were centrifuged (Z206A, Hermle, Germany) for 15 min at 4427 × g, and the supernatants containing the fat were removed. The defatted biscuits were dried at room temperature overnight. Two‐gram defatted sample was sonicated with 15 mL of 50% ethanol acidified with 0.5% HCl in an ultrasound bath (UC002BM1, Tesla, Czechoslovakia) at maximum power (300 W) for 30 min. These extracts were used for total polyphenol content (TPC), total flavonoid content (TFC), total monomer anthocyanin (TMA) content, and 2,2‐diphenyl‐1‐picrylhydrazyl (DPPH) radical scavenging activity measurements.

### 2.5 Physical parameters

The physical evaluation of biscuits was characterized in terms of thickness and diameter using a caliper on a set of six randomly selected samples as described by Drakos et al. (2016). In addition, we also examined the weight and spread ratio (diameter divided by thickness) of the biscuits (Adeola & Ohizua, 2018). The baking loss (%) was calculated based on the weight of the biscuits before and after baking.

### 2.6 Chemical analysis

All measurements were done by a Spectroquant Pharo 100 (Merck, Germany) spectrophotometer.

#### 2.6.1 Total phenolic content (TPC)

TPC was evaluated according to the method reported by Singleton and Rossi (1965) with a slight modification. Briefly, 50 μL of undiluted extracts was used, which were placed in a test tube and mixed with 1.5 mL of high‐purity water, 2.5 mL of Folin–Ciocalteu reagent (10 v/v%), and 2 mL of sodium carbonate solution (7.5 g/100 mL). The mixtures were incubated at room temperature for 90 min, and the absorbance was measured at 725 nm against the blank. The results were expressed as milligrams of gallic acid equivalents per 100 grams of dry weight of biscuit (mg GA/100 g).

#### 2.6.2 Total flavonoid content (TFC)

The TFC was assessed using a method proposed by Assefa et al. (2016). For the reaction, 0.5 mL of undiluted biscuit extracts, 1.5 mL of ethanol, 100 μL of aluminum chloride (10%), 100 μL of sodium‐acetate (1 M), and 2.8 mL of high‐purity water were reacted at room temperature for 30 min. The absorbance was read at 415 nm versus the blank, and the results were expressed as milligrams of quercetin equivalents per 100 grams of dry weight of biscuit (mg QE/100 g).

#### 2.6.3 Total monomer anthocyanin (TMA)

The pH differential protocol was used for TMA analysis (Lee et al., 2005). In brief, 0.5 mL of biscuit extract was mixed with 4.5 mL of potassium chloride (25 mM, pH = 1.0) and sodium acetate buffer (0.4 M, pH = 4.5), respectively. Each mixture was reacted at room temperature for 15 min, and the absorbance was read at two different wavelengths (520 and 700 nm) against high‐purity water. The results were expressed as milligrams of cyanidin 3‐glucoside equivalents per 100 grams of dry weight of biscuits (mg CGE/100 g), as described in our previous work (Sik et al., 2022).

#### 2.6.4 2,2‐diphenyl‐1‐picrylhydrazyl (DPPH) radical scavenging activity

The DPPH radical scavenging method was performed as described by DiNardo et al. (2019), with some modifications. A 100‐μL aliquot of diluted biscuit extract and 3000 μL of methanolic DPPH solution (0.1 mM) were reacted at room temperature for 30 min, and the absorbance was measured at 517 nm against methanol. The results were expressed as milligrams of ascorbic acid equivalents per 100 grams of dry weight of biscuits (mg AAE/100 g), as described in our previous work. Additionally, the half‐inhibitory concentration (IC_50_) was obtained through linear regression analysis interpolation.

### 2.7 Consumer acceptance

The sensory characteristics of the biscuit samples were evaluated using 12 untrained tasters (seven males and five females). The ages of the participants ranged between 23 and 54. The sensory attributes were evaluated for desirability in appearance, color, texture, taste, flavor, and overall acceptability using a 5‐point hedonic scale (5 meant they liked the samples very much, 1 meant they disliked it very much, 3 meant they neither liked nor disliked the biscuits).

### 2.8 Statistical analysis

One‐way analysis of variance (ANOVA) followed by Tukey's multiple comparison test was used to compare the significant difference (*p* < .05) in the data. All measurements were performed in triplicate. The final results were given as the mean ± standard deviation (SD).

## 3 RESULTS AND DISCUSSION

### 3.1 Physical characteristics

Table 1 presents the physical properties of biscuits fortified with edible flower petal powders. There was a decrease in the weight of the fortified samples; however, no significant differences were shown between the different concentrations (2.5 and 5.0%) used for supplementation. In the case of L_5.0_, R_2.5_, and R_5.0_ samples, the thickness was also decreased. This phenomenon can be explained by the fact that wheat flour has lower hygroscopic properties compared to dried plant powders. This allows more water to be present for gluten proteins to produce a network and increase the height of biscuits (Ramashia et al., 2021). Similar results were reported by Hernández‐Nava et al. (2023) for biscuits enriched with roselle calyx powder at different concentrations (5%, 10%, and 15%). The diameters of the biscuits ranged between 4.69 ± 0.04 and 4.78 ± 0.15 cm. Overall, these measured values showed no significant difference. The spread ratio is an important quality attribute of biscuits because of its relationship with texture, grain finesse, bite, and overall mouthfeel (Bakare et al., 2020). As shown in Table 1, the fortification step had a significant impact on this parameter. The lowest value (5.00 ± 0.26) was recorded for the control sample (C), while the highest value (6.15 ± 0.14) was observed in the case of L_2.5_, followed by R_5.0_. The spread ratio of the C, M_5.0_, and R_2.5_ was not significantly different from each other. In general, the higher spread ratio of the biscuits is more desirable (Kohli et al., 2023). Based on this fact and our results, it can be said that the supplementation of edible flower powders may increase the quality of biscuits.

**TABLE 1:** Physical characteristics of the biscuits.

Samples	Weight (g)	Diameter (cm)	Thickness (cm)	Spread ratio	Baking loss (%)
C	13.7 ± 0.82^a^	4.69 ± 0.04^a^	0.93 ± 0.02^a^	5.00 ± 0.26^a^	12.4 ± 0.61^a^
M_2.5_	11.5 ± 0.55^b^	4.81 ± 0.13^a^	0.90 ± 0.01^a^	5.34 ± 0.19^b^	14.8 ± 0.41^b^
M_5.0_	11.2 ± 0.41^b^	4.68 ± 0.21^a^	0.94 ± 0.01^a^	5.04 ± 0.08^a^	13.4 ± 0.52^b^
L_2.5_	10.3 ± 0.52^c^	4.78 ± 0.15^a^	0.78 ± 0.03^b^	6.15 ± 0.14^c^	14.6 ± 0.39^b^
L_5.0_	10.0 ± 0.63^c^	4.74 ± 0.15^a^	0.85 ± 0.01^c^	5.60 ± 0.11^d^	13.3 ± 0.52^b^
R_2.5_	11.7 ± 0.52^b^	4.59 ± 0.14^b^	0.91 ± 0.06^a^	5.05 ± 0.27^a^	14.5 ± 0.63^b^
R_5.0_	11.0 ± 0.63^b^	4.70 ± 0.16^a^	0.83 ± 0.02^c^	5.73 ± 0.25^d^	15.5 ± 0.49^b^

*Note*: Different letters indicate significant differences.

Table 1 also shows that the baking loss (%) of biscuits ranges from 12.4 ± 0.61 to 15.5 ± 0.49. The control sample has the lowest baking loss properties, while the R_5.0_ showed the highest. Djeghim et al. (2021) reported that the weight loss of gluten‐free bread increased with increasing levels of different dried fruit and vegetable by‐products due to their water‐holding capacity. From this, we can conclude that at higher plant material supplementation, the water cannot be retained in the bakery products.

### 3.2 Antioxidant properties

The evaluations of TPC, TFC, and TMA yield, as well as DPPH radical scavenging activity in the biscuits, are shown in Table 2. TPC content of the biscuits ranged between 94.5 ± 3.22 mg GAE/100 g and 715 ± 16.3 mg GAE/ 100 g. As seen in Table 2, the fortification increased the TPC in all cases and the highest increase was observed for biscuits fortified with rose petals (7 fold, from 94.5 ± 3.22 mg GAE/ 100 g to 715 ± 16.3 mg GAE/100 g) at a concentration of 5.0%. In addition, increasing the concentration of edible flower powders resulted in a considerable increase in TPC. Overall, our results were in good agreement with Hnin et al. (2020) who found that TPC in fortified cookies with rose flower powder increased with increasing incorporation. However, they found a similar increase (7.7‐fold) with higher rose flower powder (10%) supplementation, which can be explained by several methodical differences. For instance, the authors used a microwave–vacuum drying method, which took 75 min at 60°C. Furthermore, the authors used eggs for cookie preparation. The other main difference was that the authors extracted the cookies with 80% methanol at a high temperature (80°C) for 30 min without a defatting process.

**TABLE 2:** Effect of edible flowers incorporation into biscuits at different concentrations on the antioxidant properties.

Samples	TPC (mg GAE/100 g)	TFC (mg QE/100 g)	TMA (mg CGE/100 g)	DPPH (mg AAE/100 g)	IC_50_ (mg/mL)
C	94.5 ± 3.22^a^	16.8 ± 0.34^a^	n.d.	112 ± 0.27^a^	3.84 ± 0.27^a^
M_2.5_	143 ± 4.30^b^	53.1 ± 1.25^b^	n.d.	117 ± 1.05^ab^	3.66 ± 0.04^b^
M_5.0_	182 ± 1.88^c^	66.4 ± 0.83^c^	n.d.	125 ± 0.82^c^	3.73 ± 0.03^c^
L_2.5_	179 ± 6.51^c^	65.7 ± 2.91^c^	n.d.	120 ± 1.72^b^	3.54 ± 0.04^d^
L_5.0_	276 ± 13.3^d^	64.6 ± 2.29^c^	n.d.	135 ± 1.32^d^	3.16 ± 0.01^e^
R_2.5_	382 ± 16.7^e^	103 ± 4.93^d^	14.7 ± 0.89^a^	365 ± 2.57^e^	1.21 ± 0.27^f^
R_5.0_	715 ± 16.3^f^	163 ± 3.03^e^	68.1 ± 4.51^b^	546 ± 10.5^f^	0.75 ± 0.27^g^

*Note*: Different letters indicate significant differences. n.d. means not detected.

The measured yields of TFC in fortified biscuits ranged from 53.1 ± 1.25 mg QE/100 g to 163 ± 3.03 mg QE/100 g. Although we did not find any significant difference between M_5.0_, L_2.5_, and L_5.0_ samples, the TFC yields were higher in fortified biscuits compared to the control (16.8 ± 0.34 mg QE/100 g). As previously reported by several authors (Adaszyńska‐Skwirzyńska & Dzięcioł, 2017; Phuseerit et al., 2021; Wan et al., 2019), this phenomenon is presumably due to flavonoid components such as quercetin, kaempferol, apigenin, or luteolin found in edible flowers. It is well known that flavonoids have different subclasses, including anthocyanins, flavonols, flavone, and others. For this reason, in the case of roses, the higher TFC is also connected to anthocyanins, such as pelargonidin or cyanidin (Wan et al., 2019). Anthocyanins are red, blue, or purple colored pigments found in plants. According to this, TMA was only detected in biscuits fortified with rose powder. When the incorporation of rose petal powder was raised from 2.5% to 5%, the TMA significantly increased from 14.7 ± 0.89 mg CGE/100 g to 68.1 ± 4.51 mg CGE/100 g in biscuits. This was also good agreement with several studies that observed an increase in TMA content through the incorporation step compared to control cookies. For instance, this was observed when using rose petals (Hnin et al., 2020), grape pomace (Theagarajan et al., 2019), or raspberry and blueberry pomace (Šarić et al., 2016).

DPPH radical scavenging activities ranged from 117 ± 1.05 mg AAE/100 g to 546 ± 10.5 mg AAE/100 g in fortified samples and were 112 ± 0.27 mg AAE/100 g in the control biscuit. Overall, the fortified biscuits had significantly higher DPPH radical scavenging activities, except for the M_2.5_ samples (117 ± 1.05 mg AAE/100 g) compared to the control biscuit. These results are consistent with those reported by Podgórska‐Kryszczuk and Pankiewicz (2023), in which fortification of marigold water extract into bread significantly improved the DPPH radical scavenging activities. The scavenging effects of biscuits on the DPPH radical were expressed as IC_50_ values. The fortified biscuits showed significant activity when compared to values obtained for the control biscuit. Among the fortified samples, R_5.0_ exhibited the strongest activity with an IC_50_ value of 0.75 ± 0.27 mg/mL. Reduced antioxidant activity was observed in the marigold biscuits (3.66 ± 0.041 and 3.73 ± 0.031 mg/mL for M_2.5_ and M_5.0_, respectively).

Although our study was limited to baked biscuits, it is important to note that the thermal process (e.g., cooking) may significantly change the phytonutrient content of plants. This fact was supported by Castañeda‐Rodríguez et al. (2023), who evaluated the effect of cooking methods (stir‐frying, boiling, and steaming) on the biochemical compositions of agave flowers. The authors found that all cooking methods decreased up to 60% of the bioactive compounds and antioxidant capacity of agave flowers.

### 3.3 Consumer acceptability

**FIGURE 2:**
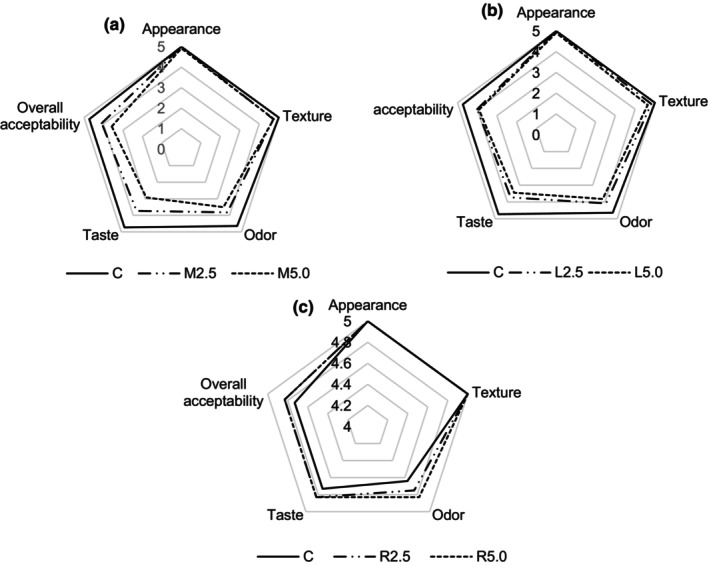
Consumer acceptance evaluation of biscuits fortified with marigold (Figure 2a), lavender (Figure 2b), and rose (Figure 2c) compared to the control (C).

The consumer acceptability evaluation on different levels of edible flower powders in biscuits, compared to the control sample, is shown in Figure 2a–c. As seen in Figure 2a, the addition of marigold petals at a concentration of 5.0% for the biscuits resulted in the lowest acceptability values for odor (3.36 ± 0.92) and taste (2.73 ± 0.79) attributes. Similar results were obtained for the M_2.5_ sample. Benvenuti et al. (2016) analyzed the sensory properties of various edible flowers and reported that marigold was the only species whose flowers were quite difficult to masticate. Moreover, the tasters found that marigold flowers are spiciness and notably bitter. Similar to marigold petals, lavender flowers, especially when used at higher concentrations (5.0%), significantly reduced the taste (3.45 ± 1.13) and odor (3.82 ± 0.98) properties of the biscuits, resulting in a decreased overall acceptability (3.91 ± 0.83) score. Demasi et al. (2021) showed that the sensory profile of *L. angustifolia* reveals a medium chewiness, bitter taste, and a specific flower aroma. It can also be found that a higher addition value significantly decreases the texture, odor, taste, and overall acceptability attributes of the biscuits fortified with marigold petals (Figure 2a) and lavender flowers (Figure 2b). Generally, the results revealed that fortification with rose petals was equal to or better than the control biscuit in almost all investigated sensory attributes (Figure 2c). For instance, there were no significant differences in appearance and texture preference scores. The values obtained for both attributes were 5.00 for fortified biscuits as well as the control. In addition, the tasters did not find any differences in the case of taste and overall acceptability for R_2.5_ and R_5.0_ biscuits. Overall, consumer acceptance was highest in the case of rose biscuits, probably due to the sweet and floral flavor of rose petals. Caser and Scariot (2022) analyzed the composition of volatile organic compounds emitted by garden rose petals and found that different components can be associated with different scents. For instance, species with high hexanal content can be associated with sweet, green, and apple scents. Varieties with high acetaldehyde content are characterized by a fruity and pungent aroma. In addition, the authors associated the 2‐phenylethanol component with a related floral and rose scent. All in all, our results were in agreement with Hnin et al. (2020) who found that the addition of rose flower powder at a concentration of 5% resulted in the highest overall acceptability score of biscuits. In the development of functional foods, a key role is played by finding ingredients that allow specific beneficial effects to be achieved without modifying the consumer acceptability of the enriched products (Podgórska‐Kryszczuk & Pankiewicz, 2023). According to this fact, our results indicate that rose petals are very promising for further research and development of functional foods.

## 4 CONCLUSION

Our study demonstrated the potential use of edible flowers as antioxidant ingredients in biscuits. From both a chemical point of view and consumer acceptability, fortification with rose petals caused remarkable changes. In all cases, the addition of different levels of edible flower powders to biscuits caused an increase in TPC, TFC, and antioxidant properties. Additionally, in the case of biscuits fortified with rose petals, the TMA contents significantly increased. The addition of edible flowers in biscuits significantly changed their spread ratio characteristics; however, the other physical properties of the biscuits were similar to the control. Taking into account the results of consumer acceptance, it is clear that marigold petals and lavender flowers make the biscuits less desirable for consumers. For both flowers, we found decreased scores for texture, odor, taste, and overall acceptability attributes. These results revealed that the biscuits fortified with rose petals showed the best antioxidant properties and consumer acceptance compared to other investigated biscuits, indicating that rose petals can be used as potential ingredients in functional foods.

## AUTHOR CONTRIBUTIONS

**Beatrix Sik:** Conceptualization (equal); methodology (equal); supervision (equal); writing – review and editing (equal). **Zsolt Ajtony:** Project administration (equal). **Erika Lakatos:** Project administration (equal). **Laura Hanna Gál:** Investigation (equal). **Rita Székelyhidi:** Project administration (equal).

## FUNDING INFORMATION

This research did not receive any specific grant from funding agencies in the public, commercial, or not‐for‐profit sectors.

## Data Availability

The data that support the findings of this study are available from the corresponding author upon reasonable request.
